# Clinical Features and Drug Characteristics of Patients with Generalized Fixed Drug Eruption in the West of Iran (2005–2014)

**DOI:** 10.1155/2015/236703

**Published:** 2015-12-10

**Authors:** Hossein Kavoussi, Mansour Rezaei, Katayoun Derakhshandeh, Alireza Moradi, Ali Ebrahimi, Harif Rashidian, Reza Kavoussi

**Affiliations:** ^1^Dermatology Department, Imam Reza Hospital, Kermanshah University of Medical Sciences (KUMS), Kermanshah 6714415333, Iran; ^2^Health School, Family Health Research Center of Kermanshah University of Medical Sciences (KUMS), Kermanshah 6714415333, Iran; ^3^Department of Pharmaceutics, School of Pharmacy, Hamadan University of Medical Sciences, Hamadan, Iran; ^4^Kermanshah University of Medical Sciences (KUMS), Kermanshah 6714415333, Iran

## Abstract

*Background.* Generalized fixed drug eruption is a specific variant of fixed drug eruption with multifocal lesions. Diagnosis of this drug reaction is straightforward, but occasionally recognition of the causative drug is not possible. This study was aimed at evaluating the clinical features and culprit drugs in generalized fixed drug eruptions in the west of Iran.* Method.* This cross-sectional study was carried out on 30 patients with criteria of generalized fixed drug eruption over 9 years. Demographic, clinical, and drug intake information were collected.* Results.* Out of 30 patients (17 females and 13 males) with the mean age of 26.67 ± 10.21 years, 28 (93.3%) and 2 (6.7%) cases had plaque and bullous clinical presentation, respectively. Upper limbs were the most common (90%) site of involvement. The antibiotic group, especially cotrimoxazole (26.1%), was reported to be the most common offending drug, but the causative drug was not determined in 7 (23.3%) patients.* Conclusion.* Many cases of generalized fixed drug eruption firstly presented as limited lesions and led to generalized lesion due to repeated intake of the causative drug. No causative drug was found in some patients, which might be associated with concurrent intake of several drugs, multiple FDE, and peculiarity of the patch test.

## 1. Introduction

Fixed drug eruption (FDE) is a specific drug reaction which characteristically recurs in the same location after reexposure to the same or related medications [1–4].

FDE is a common type of drug eruption whose incidence has tended to increase in the recent years [4].

Although diagnosis of FDE is easy for dermatologists, recognition of the offending drugs may be problematic [3, 4].

Generalized fixed drug eruption (GFDE) is a clinical variant of FDE that is presented with numerous multifocal lesions. Skin lesion is usually manifested with well-defined erythematous to violaceous round or oval plaque as generalized nonbullous fixed drug eruption and is occasionally vesicular or bullous as generalized bullous fixed drug eruption [5–7].

GFDE is not often fatal but sometimes results in cosmetic problems [1, 4, 7].

In this study, the patients with GFDE were evaluated to identify the caustic drugs, clinical type, number of episodes, location of lesions, and some demographic data of patients.

## 2. Method and Material

This cross-sectional (descriptive-analytic) study was done at Imam Reza referral hospital of Kermanshah University of Medical Sciences over 9 years, from 2005 to 2014.

The patients with typical clinical manifestation or histopathological documentation for FDE were enrolled in this study. Also, the patients had to suffer from at least three involved locations and a minimum of 10 lesions.

The offending drugs were identified by taking a meticulous history or performing the patch test at the site of previous FDE lesions with the medications consumed over the last 30 days.

Demographic data such as age, gender, history of medications, number of episodes, clinical features, and causative drug were recorded.

Analysis of data was carried out by SPSS (version 22) software. Analysis of qualitative data was done by Chi-square and Fisher's exact test. For quantitative variables, firstly, one sample KS test was used to measure the normality and later was applied based on the results of Leven's and independent *t*-test or Mann-Whitney *U* test. Significance level was considered 0.05 for analysis of the tests.

## 3. Results

A total of 30 patients, 17 (56.7%) females and 13 (43.3%) males (Table 1), were recruited in this study.

The age range of the patients was 13–57 years with the mean age of 26.67 ± 10.21 years. Fourteen (46.7%) patients stood in the third decade. The mean age in bullous variant (44.5 ± 17.8) was more than that of nonbullous variant (25.39 ± 9.78) of GFDE, but there was no statistically significant difference between them.

Most of the patients (73.3%) were diagnosed by their typical clinical manifestation, but 27.8% of patients were diagnosed through histopathologic documentation.

The range of FDE episode was 1–10 times with the mean of 3.77 ± 2.11. Most of the subjects (39%) had 3 episodes (Table 1). The number of episodes was more in females and nonbullous GFDE. There was a significant difference between episode and gender (PV = 0.043) but not clinical features (PV = 0.225).

Involvement of the upper limbs, as the most common location of distribution, was seen in 27 (90%) cases. GFDE lesions were located on lower limbs in 21 (70%) cases, on trunk in 20 (66.7%) cases, and on face and scalp in 10 (33.3%) cases (Table 1).

FDE lesions were seen in 9 (30%) and 8 (26.7%) cases in genital and oral mucosa areas, respectively (Table 1).

As for the clinical presentation, 28 (93.3%) patients had plaque skin lesions, while bullous lesions were found in 2 (6.7%) cases (Table 1).

There was no significant difference between clinical features and gender (PV = 0.492), multiple drug intake (PV = 0.1), and mucous membrane involvement (PV = 0.492).

When the patients were referred to the clinic, multidrug consumption was reported in 25 (83.3%) patients, but only 5 (16.7%) of them had monodrug intake (Table 2).

Although recognition of the offending drugs was distinguished in 23 (76.6%) cases, it was not distinguished in 7 (23.3%) patients (Table 2). Patch test was not positive in any patients with medication intake in the last 30 days.

The category of causative drugs included 14 (60.9%) antibiotics, 7 (30.4%) analgesics, and 2 (8.7%) miscellaneous cases. The most common offending drugs were cotrimoxazole in 6 (26.1%) cases, metronidazole in 4 (17.4%) cases, and ibuprofen in 3 (13%) cases (Table 2). GBFDE was seen in two females, one due to metronidazole and the other due to cotrimoxazole.

## 4. Discussion

Our findings indicated that the majority of patients with GFDE were females with concurrent multidrug intake, involvement of upper limbs, experience of several episodes, and antibiotic intake, especially cotrimoxazole.

Our results showed most of the patients were females in the third decade of their life. Jung et al. [3] and Ognongo-Ibiaho and Atanda [8] reported a higher frequency of FDE for males in their fourth decade of life, but Mahboob and Haroon [9] found equal rates in both sexes.

In a study from Taiwan [10], most patients had previous events of FDE. Also, Jung et al. [3] found a mean episode of 2.6 in their patients. In our patients, the high frequency of episode may be related to the existence of atypical cases of FDE and unfamiliarity of doctors of medicine, especially general practitioners, with FDE.

We performed biopsy and histopathologic evaluation in some patients for definite diagnosis because of atypical forms of FDE such as GBFDE [5–7] and nonpigmented variant [11] that required histopathologic documentation.

FDE sometimes presents with a solitary lesion. However, with reuse of causative drugs and induction of new attack, in addition to previous lesions, new lesions appear [1, 3, 4, 9–11]. In some studies, extremities have been reported as the most common site of involvement [1, 3, 11]. Also, Lee et al. [10] reported upper extremities as the most common involved location. In our patients, consistent with previous studies, the lesions were mostly located on upper extremities.

In FDE, genital area involvement is most frequently caused by cotrimoxazole [12]. However, involvement of penis is uncommon [13] and a retrospective study showed, from 321 cases, 16.5% of them had genital area involvement [14]. High frequency of genital area involvement (30%) in our study was related to the presence of bullous variant (6.7%), cotrimoxazole as the most common causative medication (26.1%), and multiple attacks in our patients.

Mucous membrane FDE lesions may be alone or in association with skin involvement [15], but mucous membrane involvement is more common in the bullous variant than in nonbullous variant of GFDE (66.7% versus 30%) [10]. Twenty-six percent of our patients showed erosion in oral mucous membrane, which is consistent with other results of studies.

The patients with GFDE usually take multiple drugs at a time [10, 16], which is consistent with the results of our study. We tend to think that, in drug reactions, taking multiple drugs concurrently is one of the problems that impede the accurate recognition of the causative drug.

We could not determine the offending drug in approximately one-fourth of the patients. Offending drug was not recognized by Lee [4] and Lee et al. [10] in 71.6% and 23% of patients, respectively. Concurrent intake of multiple drugs [10, 16], multiple FDE [17], controversial usefulness of patch test [18], self-medication, and inaccurate past medical history reported by the patients [19] are the most important impediments for determination of culprit drugs.

In most studies, multifocal or generalized FDE is not common [4, 10, 16], and bullous type of GFDE is its rare form, which must be differentiated from Stevens-Johnson syndrome and toxic epidermal necrolysis [7, 10]. Repeated intake of causative drug or related drugs, in addition to old lesions, induces new lesions which occasionally become generalized [1, 19]. We think dissemination in most of our cases may be associated with atypical presentation, unawareness of FDE on the part of general practitioners, self-medication, and confusing history reported by the patients, which resulted in delay in diagnosis and avoidance of culprit drug.

Consistent with several studies [9, 14, 20, 21], our findings showed antibiotic drugs, especially cotrimoxazole, were the most common culprit drugs in FDE. But in other studies, analgesics medications [2, 3, 22] have been frequently reported as offending drugs in this type of drug reaction. In a case series by Patro et al. [16] and another study by Lee et al. [10], recognition of incremental drug was not possible in most of the patients with multifocal FDE or GFDE because of multiple drug intake. The difference of causative drugs in various areas may be dependent on self-medication in the community [20], genetic predisposition [23], concurrent intake of multiple drugs [16], and popular drugs in each region [19].

In conclusion, it seems that many cases of GFDE other than bullous GFDE are first presented as FDE with limited lesions, which result in disseminated FDE by repeated exposure to the causative drug. The most common offending drug belonged to antibiotic group, especially cotrimoxazole, and the most involved site was reported as the upper limb. Sometimes the causative drug is not recognized, which may be due to concurrent intake of several drugs, multiple FDE, and absence of distinctive paraclinic assessment for recognition of causative drugs. We suggest detailed education of medical students about drug reactions, especially FDE, multiple FDE as a unique drug reaction during concurrent intake of several drugs, and obtaining a detailed history of patients to determine the offending drug.

## Figures and Tables

**Table 1:** Demographic data and clinical features of patients with generalized fixed drug eruption.

Variables	
Mean of age (years)	26.67 ± 10.21
Gender (F/M)	17/13
Mean of episode	3.77 ± 2.11
Location	
Face and head (*n*%)	10 (33.3%)
Upper limbs (*n*%)	27 (90.0%)
Trunk (*n*%)	20 (66.7%)
Lower limbs (*n*%)	21 (70.0%)
Genital area (*n*%)	9 (30.0%)
Mucous membrane (*n*%)	8 (26.7%)
Clinical feature	
Nonbullous (*n*% )	28 (93.3%)
Bullous (*n*%)	2 (6.7%)

**Table 2:** Drug behavior and offending drugs.

Behavior of drug intake	
Monodrug intake (*n*%)	5 (16.7%)
Multiple drug intake (*n*%)	25 (83.3%)
Recognized caustic drug (*n*%)	23 (73.3%)
Antibiotic (*n*%)	14 (60. 9%)
Cotrimoxazole (*n*%)	6 (26.1%)
Metronidazole (*n*%)	4 (17.4%)
Doxicyclin (*n*%)	2 (8.7%)
Azithromycin (*n*%)	1 (4.3%)
Nitrofurantoin (*n*%)	1 (4.3%)
Analgesic (*n*%)	7 (30.4%)
Ibuprofen (*n*%)	3 (13.0%)
Mefenamic acid (*n*%)	1 (3.4%)
Meloxicam (*n*%)	1 (3.4%)
Novafen (*n*%)	1 (3.4%)
Miscellaneous (*n*%)	2 (8.7%)
Unrecognized caustic drug (*n*%)	7 (26.7%)

## References

[1] Breathnach S. M., Burns T., Breathnach S., Cox N., Griffiths C. (2010). Drug reactions. *Rook's Textbook of Dermatology*.

[2] Heng Y. K., Yew Y. W., Lim D. S. Y., Lim Y. L. (2015). An update of fixed drug eruptions in Singapore. *Journal of the European Academy of Dermatology and Venereology*.

[3] Jung J.-W., Cho S.-H., Kim K.-H., Min K.-U., Kang H.-R. (2014). Clinical features of fixed drug eruption at a tertiary hospital in Korea. *Allergy, Asthma and Immunology Research*.

[4] Lee A.-Y. (2000). Fixed drug eruptions. Incidence, recognition, and avoidance. *American Journal of Clinical Dermatology*.

[5] Sawada Y., Nakamura M., Tokura Y. (2011). Generalized fixed drug eruption caused by pazufloxacin. *Acta Dermato-Venereologica*.

[6] Can C., Akkelle E., Bay B., Arıcan Ö., Yalçın Ö., Yazicioglu M. (2014). Generalized fixed drug eruption in a child due to trimethoprim/sulfamethoxazole. *Pediatric Allergy and Immunology*.

[7] Lipowicz S., Sekula P., Ingen-Housz-Oro S. (2013). Prognosis of generalized bullous fixed drug eruption: comparison with Stevens-Johnson syndrome and toxic epidermal necrolysis. *British Journal of Dermatology*.

[8] Ognongo-Ibiaho A. N., Atanda H. L. (2012). Epidemiological study of fixed drug eruption in Pointe-Noire. *International Journal of Dermatology*.

[9] Mahboob A., Haroon T. S. (1998). Drugs causing fixed eruptions: a study of 450 cases. *International Journal of Dermatology*.

[10] Lee C.-H., Chen Y.-C., Cho Y.-T., Chang C.-Y., Chu C.-Y. (2012). Fixed-drug eruption: a retrospective study in a single referral center in northern Taiwan. *Dermatologica Sinica*.

[11] Shelley W. B., Shelley E. D. (1987). Nonpigmenting fixed drug eruption as a distinctive reaction pattern: examples caused by sensitivity to pseudoephedrine hydrochloride and tetrahydrozoline. *Journal of the American Academy of Dermatology*.

[12] Özkaya-Bayazit E. (2003). Specific site involvement in fixed drug eruption. *Journal of the American Academy of Dermatology*.

[13] Lawrentschuk N., Pan D., Troy A. (2006). Fixed drug eruption of the penis secondary to sulfamethoxazole-trimethoprim. *TheScientificWorldJournal*.

[14] Saka B., Kombaté K., Médougou B.-H. (2012). Fixed drug eruption in dermatology setting in Lomé (Togo): a retrospective study of 321 cases. *Bulletin de la Societe de Pathologie Exotique*.

[15] Jain V. K., Dixit V. B., Archana (1991). Fixed drug eruption of the oral mucous membrane. *Annals of Dentistry*.

[16] Patro N., Panda M., Jena M., Mishra S. (2013). Multifocal fixed drug eruptions: a case series. *International Journal of Pharmaceutical Sciences Review and Research*.

[17] Verbov J. (1985). Fixed drug eruption due to a drug combination but not to its constituents. *Dermatologica*.

[18] Ohtoshi S., Kitami Y., Sueki H., Nakada T. (2014). Utility of patch testing for patients with drug eruption. *Clinical and Experimental Dermatology*.

[19] Padmavathi S., Manimekalai K., Ambujam S. (2013). Causality, severity and preventability assessment of adverse cutaneous drug reaction: a prospective observational study in a tertiary care hospital. *Journal of Clinical and Diagnostic Research*.

[20] Sehgal V. N., Srivastava G. (2006). Fixed drug eruption (FDE): changing scenario of incriminating drugs. *International Journal of Dermatology*.

[21] Sharma R., Dogra D., Dogra N. (2015). A study of cutaneous adverse drug reactions at a tertiary center in Jammu, India. *Indian Dermatology Online Journal*.

[22] Shukla S. R. (1981). Drugs causing fixed drug eruptions. *Dermatologica*.

[23] Tohkin M., Ishiguro A., Kaniwa N., Saito Y., Kurose K., Hasegawa R. (2010). Prediction of severe adverse drug reactions using pharmacogenetic biomarkers. *Drug Metabolism and Pharmacokinetics*.

